# Tubercle of Brain; with Remarks

**Published:** 1856-12

**Authors:** William Pepper

**Affiliations:** The Physicians of the Pennsylvania Hospital


### MEDICAL EXAMINER.

NEW SERIES.—NO. CXLIV.—DECEMBER, 1856.

---

### ORIGINAL COMMUNICATIONS.

---

*Tubercle of Brain ; with Remarks.* By WILLIAM PEPPER, M.D., one of the Physicians of the Pennsylvania Hospital.

A young man, aged 18 years, entered the hospital April 3, 1856, complaining of severe paroxysmal headache. He stated that the first attack came on about three months before, and lasted only a few minutes; from this time it recurred every two weeks until the end of the second month, when the pain became more frequent and protracted, coming on every week during the afternoon, and lasting about half an hour. It was not confined to any particular locality; at times shooting through the temples, and then in the back of the head; frequently it was so severe as to cause the patient to shriek, and was then evidently attended with some confusion of ideas, though during the intervals, his intelligence was unimpared. When first admitted, the paroxysms came on four or five times a day, and were evidently not so severe or protracted. He complained of tinnitus aurium and slight numbness of the upper and lower extremities, their sensibility, however, being unimpaired. Whilst in the horizontal position he appeared to have perfect control of his movements, but when attempting to walk, his gait was staggering, and he was evidently disposed to fall backwards. The pupils were occasionally dilated, but contracted under light, and the sight was apparently as good as usual; his sense of smell, taste, and hearing were also natural, nor was there the least distortion of his features, dysphagia, or other manifestations of paralysis.

Though carefully interrogated, he persisted in stating that he never had been subject to cough, pain, expectoration, or other pulmonary symptoms; he admitted, however, that his mother had died of phthisis, and that last summer, whilst making some unusual exertion, he had thrown up about a tablespoonful of pure blood. The patient was also thin and pale, but without hectic fever; pulse variable, at times frequent, and then again slow; loss of appetite, but no vomiting; bowels constipated. Percussion under the left clavicle was dull, and in this locality the respiratory murmur was feeble and attended with some crackling and prolonged expiration; throughout the rest of the lungs nothing abnormal could be detected. It was also observed that the ends of the fingers were somewhat enlarged and the nails curved, in the manner so frequently seen in tubercular diseases. The treatment consisted chiefly in the administration of Iod. Potass. grs. v. three times a day, in connection with the occasional application of dry cups to the nucha, followed by a small blister. He was allowed a moderately nutritious, but not stimulating diet; and to relieve the paroxysms took Ext. Hyoscyam. grs. iii. three times a day. No important change occurred up to the 16th of the month, when for the first time he was seized with convulsions, and immediately expired, just two weeks after his admission into the hospital.

On examining the body the following day, the membranes of the brain presented nothing unusual, and the cerebrum was of good consistence, and in all respects perfectly natural. The cerebellum, however, was much softer than usual throughout, but especially so at the central portions of both hemispheres; and a large encysted tumor occupied the posterior part of its right lobe, but also encroached more or less upon the left side, and was attached to the pia-mater by loose cellular tissue. The mass was spheroidal, and measured about an inch and half in its longest diameter; it was composed of firm yellowish-gray tubercular matter—such as is often found in the mesenteric glands—contained in a tough fibrous cyst about as thick as ordinary parchment. The apex of left lung was adherent, and somewhat indurated by tuberculous deposit in various stages of advancement, the rest of the lung being perfectly healthy; a few granular tubercles were disseminated throughout upper lobe of right lung. Other organs natural.

The above case appears interesting and worthy of record as illustrating in a striking manner the well known fact, that tumors of considerable dimensions may be imbedded in the brain without materially interfering with the functions of that organ. From the size and character of the mass, it is clear that it must have existed for many months, and was probably long antecedent to the paroxysms of pain, which, until a few days previous to death, formed the only manifestation of diseased action. It is also worthy of remark that the pain was not only intermittent, but strictly periodical, and therefore well calculated to mislead as to its real cause. Most writers dwell upon this peculiarity in affections of the brain, but it is not often that a case so strongly illustrative of this fact, falls under observation. By a singular coincidence, the previous occupant of the same bed died with central softening of the cerebrum, and presented a very similar train of symptoms. Here, too, the pain was intermittent, and at times more or less periodical, though not to the same extent as in the case just reported; and yet, sufficiently so to have induced his former physician to administer quinia in full does, arsenic, belladonna, aconite and other anti-intermittent and neuralgic remedies. What is not a little remarkable in this case, is the circumstance that the paroxysms of pain, though clearly the result of inflammatory action, were nevertheless in a measure postponed and mitigated by the *quinine treatment.* Quite recently the writer saw in consultation with Dr. Morris, a remarkable instance of cancerous tumor, but little less than a pullet's egg, imbedded in the central portion of left hemisphere of cerebrum; and yet, until a few days before death, unattended with delirium, paralysis or convulsions. The patient, it is true, had been for several years subject to violent sick headache, coming on at irregular periods; but presenting nothing peculiar or in any way calculated to excite suspicion as to the existence of organic disease of the brain. And indeed it was nothing but the cancerous cachexia, in connection with a deep seated tumor in the pelvis, that enabled his physicians to predict a similar degeneration in the brain, even after cerebral symptoms had declared themselves.

In like manner in the case under consideration, it was not until the tinnitus aurium, numbness, dilated pupil, &c., had supervened, in connection with the knowledge of the tubercular affection of the lungs, as manifested by the physical signs, that the real nature of the brain affection was fully comprehended. In this connection it may not be uninteresting to allude to the latent nature of the pulmonary disease as associated with and influenced by the cerebral tumor. It will be observed that the patient had neither cough, expectoration, dyspnœa, or other symptom indicative of disease of the lungs; and we cannot but think that this peculiarity was owing to the diseased condition of the brain rendering it insusceptible to the impressions transmitted from the suffering organs. On the same principle, we frequently find even acute and extensive pleuro-pneumonia perfectly latent. It is no uncommon thing for patients to enter the wards suffering with fever and delirium, but entirely uncomplicated by cough, expectoration, pain or oppression; and yet, auscultation and percussion revealing in these cases extensive inflammation of the lungs; and it is interesting to observe that just in proportion as the brain symptoms subside, the cough, rusty sputa, pain and oppression are developed. So much so indeed is this the case, that the writer is constantly in the habit of making a careful exploration of the chest in all cases of obscure cerebral affection; and he has certainly thus been enabled to form a correct diagnosis, and consequently to institute a rational treatment in many cases, which must otherwise have been entirely misunderstood. Owing to a neglect of this precaution, it occasionally happens that pneumonia or phthisis in the insane steals a march upon the medical attendant, or even runs on to a fatal result, without having awakened the least suspicion as to the real state of the case. Now although in the case above reported the symptoms were not the same as in the instances just referred to, yet the analogy is sufficiently close to justify the belief that the tubercular affection of the lungs was masked by the accompanying cerebral complication; it is true there was no marked delirium, yet it is equally clear that the general sensibility was greatly obtunded.

In conclusion, it may be observed that the pathological appearances presented nothing very unusual, since most writers upon diseases of the brain allude to instances of a somewhat similar character. Abercrombie reports the case of a child only three years old, in which there were "two tubercles the size of large walnuts in the posterior part of the cerebellum;" and another case of a lad aged seven years, who until a short time before death presented no cerebral symptoms, and yet had imbedded in the right hemisphere a tubercular mass "nearly five inches in circumference." The present case, however, appears especially interesting, not only as showing the perfectly paroxysmal character of the pain which occasionally attends under these circumstances, but also as affording an instance of a latent tumor of the brain tending to mask a serious concomitant disease of the lungs.

